# Gut Microbial Metabolism as a Dynamic Interface in Neurodegenerative Diseases

**DOI:** 10.3390/metabo16090642

**Published:** 2026-09-02

**Authors:** Zhuangxiu Kang, Ran Meng, Meng Nie, Tianqi Wang

**Affiliations:** 1 School of Public Health, Capital Medical University, Beijing 100054, China; 2 School of Traditional Chinese Medicine, Capital Medical University, Beijing 100054, China

**Keywords:** gut–brain axis, microbial metabolites, neurodegenerative diseases, Alzheimer’s disease, APOE4

## Abstract

Gut microbial metabolism links intestinal ecology with systemic physiology and neural pathology, but its effects vary across disease stage, tissue compartment, and host background. This review uses Alzheimer’s disease (AD) as the principal model and compares selected features with Parkinson’s disease (PD) and amyotrophic lateral sclerosis (ALS). Across the AD continuum, fermentation-related changes appear in prodromal cohorts, whereas broader alterations in amino acid products, host–microbial co-metabolites, bile acids, and lipids accompany mild cognitive impairment and dementia. These group-level patterns do not constitute a fixed patient trajectory. Microbial production, intestinal absorption, hepatic conversion, renal clearance, barrier integrity, and tissue-specific receptors jointly determine biological exposure. Experimental studies connect short-chain fatty acids and indole derivatives with epithelial and neuroimmune homeostasis, while imidazole propionate, trimethylamine N-oxide, selected kynurenine products, and remodeled bile acid pools engage vascular, inflammatory, amyloid, or tau-related pathways. Cerebral pathology can also remodel the intestinal ecosystem, creating reciprocal feedback. Apolipoprotein E4 modifies lipid handling, vascular permeability, and immune responses, helping to explain why comparable metabolic profiles may carry different consequences among individuals. Translation therefore requires more than a change in community composition. Trials must verify microbial function, metabolite target engagement, AD biomarker response, and clinical benefit in appropriately stratified participants. Shared pathways in PD and ALS provide comparison points, but disease-specific cells, proteinopathies, and treatment exposures constrain direct transfer of AD-derived targets.

## 1. Introduction

Neurodegenerative diseases place an expanding burden on ageing societies. The number of people living with dementia was estimated at 57.4 million in 2019 and is projected to reach 152.8 million by 2050, largely because of population growth and ageing [1]. Incidence has declined in some high-income cohorts, but the pattern is not geographically uniform and will not offset the rise in absolute case numbers [2]. Parkinson’s disease (PD) is also becoming more prevalent [3]. Amyotrophic lateral sclerosis (ALS) is rarer, progresses rapidly, and remains difficult to quantify when registry coverage is limited [4]. These disorders affect different neural systems and express distinct protein pathologies, yet they converge on progressive neuronal loss, disturbed energy homeostasis, inflammation, and network failure [5]. Alzheimer’s disease (AD) provides the main clinical continuum for examining how these processes evolve before and after cognitive impairment.

Biomarkers have moved the field closer to the presymptomatic interval. Amyloid and tau imaging, cerebrospinal fluid assays, and blood measurements can detect cerebral pathology before dementia and support diagnosis, trial enrolment, and treatment monitoring [6]. α-Synuclein seed amplification assays are driving a similar shift in PD, although their prognostic value and sensitivity to progression remain under evaluation [7]. Osteopontin illustrates the importance of timing. Cerebrospinal fluid concentrations were elevated in biomarker-positive individuals without dementia and were associated with faster subsequent cognitive decline, but the association with conversion weakened after established AD biomarkers were included [8]. A measurable signal can therefore carry stage-dependent information without defining an independent disease trajectory.

Metabolic measurements add a spatial constraint. Post-mortem brain tissue provides anatomical detail at a single late time point. Cerebrospinal fluid lies closer to the central nervous system, whereas blood permits repeated sampling but integrates metabolism across organs. Paired plasma and cerebrospinal fluid profiling in AD separated systemic energy disturbances from central changes in tryptophan metabolism and creatinine handling [9]. Some peripheral signals tracked blood–brain barrier permeability, while others remained compartment-specific. Gut microbes add an accessible functional layer by transforming dietary and host substrates into short-chain fatty acids (SCFAs), tryptophan derivatives, modified bile acids, and precursors of host–microbial co-metabolites [10,11]. Absorption, hepatic conversion, renal clearance, and barrier passage then reshape the concentration measured in each compartment.

Human studies connect this metabolic capacity with AD-related phenotypes. Fecal SCFAs and propionate-producing bacteria vary with sex and amyloid status, and selected associations extend to cerebrospinal fluid markers, plasma phosphorylated tau, and cognitive change [12]. Taxonomic signatures are less portable. A large PD meta-analysis detected recurrent microbial and functional differences, yet prediction models trained in one cohort transferred poorly to others [13]. Diet, medication, geography, intestinal transit, analytical platform, and disease definition all contribute to this instability. Most AD datasets also compare different participants at one time point. Differences among cognitively unimpaired, subjective impairment, mild cognitive impairment, and dementia groups cannot be assembled into the longitudinal history of one patient.

For this narrative review, PubMed was searched from database inception to 27 August 2026. The complete search comprised three concept blocks. The microbiome block was (gut microbiota[Title/Abstract] OR gut microbiome[Title/Abstract] OR intestinal microbiota[Title/Abstract]). The metabolic block was (metabolite*[Title/Abstract] OR short-chain fatty acid*[Title/Abstract] OR SCFA*[Title/Abstract] OR tryptophan[Title/Abstract] OR indole*[Title/Abstract] OR kynurenine*[Title/Abstract] OR trimethylamine N-oxide[Title/Abstract] OR TMAO[Title/Abstract] OR bile acid*[Title/Abstract] OR imidazole propionate[Title/Abstract] OR lipid*[Title/Abstract] OR lipopolysaccharide[Title/Abstract] OR extracellular vesicle*[Title/Abstract]). The disease block was (Alzheimer Disease[MeSH Terms] OR Alzheimer*[Title/Abstract] OR Parkinson Disease[MeSH Terms] OR Parkinson*[Title/Abstract] OR Amyotrophic Lateral Sclerosis[MeSH Terms] OR amyotrophic lateral sclerosis[Title/Abstract]). The combined query joined the three blocks with AND and retrieved 1497 records. Disease-specific searches retained the microbiome and metabolic blocks and used the corresponding individual disease block. These searches retrieved 979 AD-related, 743 PD-related, and 97 ALS-related records. Because the disease-specific results overlapped, their counts were not summed.

Human, animal, and cell studies were eligible when they linked a defined microbial function, microbial metabolite, host–microbial co-metabolite, host-derived metabolite, or non-metabolite microbial product to a metabolic, pathological, biomarker, or clinical outcome. Studies reporting taxonomic differences alone were retained only when they provided essential contextual evidence. Records were managed in Zotero and checked using DOI, PMID, and title information. Titles and abstracts were assessed first, followed by full-text evaluation when the abstract did not establish a biological origin, compartment, study design, mechanism, or evidentiary strength. For the evidence synthesis, we recorded study design, disease definition or experimental model, sample characteristics, biological compartment, analytical platform, reported covariates, direction of association or intervention effect, and evidence level.

The original searches were conducted iteratively, and prospective counts for title/abstract screening and full-text assessment were not retained. The updated search documented the reproducibility and scope of the review but was not used to reconstruct an inaccurate retrospective screening flow. The final manuscript contains 177 unique cited records, including primary studies, reviews used to develop the conceptual framework, and studies identified through reference-list screening.

The resulting evidence is organized around AD as the principal disease model. Stage-associated metabolic patterns are described first, followed by the microbial and host mechanisms that may generate them, sources of interindividual variation, and the requirements for metabolic intervention. PD and ALS are then used as comparative disorders to examine which microbial metabolic features are shared across neurodegeneration and which remain disease-specific. This organization allows microbial metabolism to be considered as an interface between intestinal function, host physiology, and neural pathology without assuming that the same pathway has identical consequences across diseases.

## 2. Gut Microbial Metabolic Alterations Across AD-Related Clinical States

Subjective cognitive decline (SCD), mild cognitive impairment (MCI), and AD dementia represent clinically distinct groups, but SCD and clinically defined MCI are not equivalent to biomarker-confirmed preclinical or prodromal AD. Studies summarized in this section use heterogeneous diagnostic definitions: some classify participants by cognitive status alone, whereas others additionally require amyloid or tau evidence. The comparisons below should therefore be interpreted as group-level metabolic differences across AD-related clinical or biomarker-defined states rather than as a fixed sequence within individual patients [14]. Fermentation-related alterations have been reported in several pre-dementia cohorts, while broader changes in amino-acid products, host–microbial co-metabolites, bile acids, and lipids are increasingly described in MCI and dementia [15].

### 2.1. Pre-Dementia States

#### 2.1.1. Subjective Cognitive Decline

Direct metabolic evidence in SCD is limited. Biomarker-defined preclinical AD offers an adjacent view before objective cognitive impairment, but the two states are not interchangeable. Cognitively unimpaired adults with amyloid pathology already differ from amyloid-negative controls in microbial composition and predicted function [16]. Combining fecal microbial features with plasma amyloid measures also distinguishes these groups [17]. Neither study defines a metabolic profile unique to SCD.

Fermentation-related changes provide the clearest early signal. Paired fecal and plasma multi-omics detected cognition-associated metabolic alterations in both compartments, with fecal changes appearing before corresponding plasma differences [18]. Fecal propionate and propionate-producing bacteria varied with sex and amyloid status in another human cohort [12]. These measurements capture different biological processes. Feces contain metabolites remaining after local production and absorption. Blood concentrations additionally reflect tissue use and clearance.

Serial perioperative sampling shows how the prodromal gut responds to physiological stress. Before surgery, participants with SCD or amnestic MCI had fewer SCFA-producing bacteria and greater evidence of intestinal barrier disturbance than cognitively normal participants. Surgery widened these differences [19]. This design tests susceptibility to an acute challenge rather than natural progression from SCD to MCI.

Temporal evidence is clearer in animals. Longitudinal sampling of an AD mouse model showed that butyrate-producing bacteria and cecal butyrate declined before overt neuropathology and memory impairment [20]. Human studies place fermentation-related abnormalities near the preclinical phase. The order between microbial change, amyloid deposition, and subjective symptoms remains unresolved within individual patients.

#### 2.1.2. Mild Cognitive Impairment

MCI contains a more reproducible fermentation deficit. *Faecalibacterium prausnitzii*, a prominent butyrate producer, is depleted and correlates with cognitive performance [21]. Lower abundance of other SCFA-producing taxa accompanies higher odds of amyloid and phosphorylated tau positivity [22]. Brain imaging adds another layer. Fewer butyrate producers occur alongside greater free water in MCI and AD [23]. These studies converge on fermentation-related ecology, not on one diagnostic bacterium.

Community differences extend into microbial functions. Shotgun metagenomics identifies coordinated species and pathway alterations related to cognition and AD biomarkers [24]. A smaller comparison found substantial overlap between MCI- and AD-associated profiles [25]. Mediterranean cohorts likewise separated cognitively impaired participants from healthy aging, although the contributing taxa differed between populations [26,27]. The recurring case–control separation is more consistent than any universal taxonomic list.

Longitudinal studies narrow the possible direction of change. Microbial genes involved in the urea cycle, polyamine synthesis, and methionine or cysteine metabolism predicted poorer cognitive performance in older adults at risk of AD [28]. Baseline community features also predicted subsequent cognitive and depressive symptoms during a two-year follow-up [29]. These outcomes concern symptom change, not conversion to AD dementia.

Intestinal physiology shapes the same metabolic environment. Infrequent bowel movements were associated with fewer butyrate-producing species and poorer cognition in a large population study [30]. Bowel frequency is not a metabolite biomarker. It influences transit time and the ecological conditions under which microbial fermentation occurs.

Blood profiling adds a systemic layer at MCI. Serum tryptophan products, indoxyl sulfate, choline-related compounds, and cresol metabolites distinguished cognitively healthy adults, subjective impairment, and MCI [31]. Plasma trimethylamine N-oxide (TMAO) was higher in participants with MCI in a population with elevated cardiovascular risk [32]. The cardiovascular setting is part of that association. Direct metabolomic comparisons further identified altered SCFAs, lithocholic acid, and tryptophan products across amnestic MCI and AD [33]. The MCI profile therefore extends beyond fermentation without yielding a single stage-specific metabolite.

### 2.2. AD Dementia

In clinical AD, measured differences span the largest number of compartments. Fecal metagenomic functions differ between people with dementia and cognitively unimpaired controls, and selected features correlate with cerebrospinal fluid markers of AD pathology [34]. Blood and cerebrospinal fluid analyses add immune, proteomic, and tryptophan-related signatures linked to amyloid and tau status [35]. The observed profile is no longer confined to intestinal output.

Bile acid composition changes in a relatively consistent direction. Primary bile acids are produced in the liver, whereas gut bacteria further transform the bile acid pool into secondary species [36]. Serum analyses show lower primary cholic acid together with higher bacterially modified secondary species and ratios in AD [36]. Individual bile acids correlate with cognition, brain atrophy, glucose metabolism, and cerebrospinal fluid amyloid or phosphorylated tau [37]. Two large biomarker-defined cohorts provide longitudinal evidence. Baseline lithocholic and deoxycholic acids predicted subsequent amyloid and tau progression [38]. Recurrent redistribution of the bile acid pool is better supported than any single late-stage marker.

Other products broaden the systemic profile. Dietary trimethylamine-containing substrates are converted by gut microbial enzymes to trimethylamine (TMA). Absorbed TMA is subsequently oxidized, predominantly by hepatic flavin-containing monooxygenase 3, to trimethylamine N-oxide (TMAO). Cerebrospinal fluid TMAO is elevated in MCI and AD and correlates with AD biomarkers and neuronal injury [39]. Large-scale metabolic phenotyping links conjugated bile acids, branched-chain amino acids, glutamate-related features, and ammonia homeostasis with cognitive stage and amyloid burden [40]. Reviews report substantial variation in the direction and strength of individual gut-derived compounds [10]. TMAO and ammonia belong to a wider state of host–microbial co-metabolism.

Animal multi-omics helps connect the compartments. Established pathology in APP/PS1 mice coincides with microbiota-associated differences in fecal, serum, and cortical metabolites, including bile acids and unsaturated fatty acids [41]. Transfer of an aged AD-associated community to younger transgenic mice aggravates pathology and reproduces lipid metabolic disruption [42]. These experiments show that dysbiosis can reorganize peripheral and cerebral metabolism together. Whether humans follow the same order has not been tested longitudinally.

### 2.3. Cross-State Metabolic Patterns

Comparisons across independently defined clinical groups reveal recurring metabolic patterns, but they do not establish within-person trajectories. Microbial network organization differs among SCD, MCI, and AD even when individual taxa do not change monotonically [43]. Multi-omics analyses identify progressive links among microbial composition, fecal metabolites, brain structure, and cognition [44]. Multimodal profiles distinguish the clinical stages most effectively when microbiomic and metabolomic features are combined with imaging and clinical variables [45].

Stage-associated differences occur at several measurement levels. Across independent cohorts, fecal fermentation-related features are reported more often in SCD or biomarker-defined preclinical groups. MCI studies report microbial pathway and circulating metabolite differences, whereas dementia cohorts show wider systemic involvement. Together, these comparisons locate the relative prominence of each signal across groups. A large multi-omics study independently connects gut microbial features with cognition, hippocampal volume, serum metabolites, and inflammation, while showing that these data layers need not move in parallel [46]. Fecal and circulating measurements should therefore be treated as complementary rather than interchangeable.

Most human studies compare separate diagnostic groups. Diagnostic definitions, diet, medication, geography, sequencing methods, and metabolomic platforms vary substantially [47,48]. Several mechanistic metabolite findings remain better established in animals than in patients [49]. Small datasets and platform dependence also restrict the portability of multi-omics classifiers [50]. These design features prevent reconstruction of one person’s trajectory from SCD to dementia.

Circulating profiles contain additional host variation. Genetic background, microbial activity, lifestyle, comorbidities, and medication jointly contribute to blood metabolite concentrations [51]. Population averages can still identify a broad metabolic ordering, but they cannot assign a universal transition point. The same stage may therefore contain distinct metabolic states, and similar metabolite profiles may carry different pathological consequences. Explaining this heterogeneity requires mechanisms that connect microbial production with host conversion, barrier exposure, and tissue response.

## 3. Microbial Metabolic Mechanisms in Alzheimer’s Disease

Stage-associated profiles raise two mechanistic questions. Why do fermentation products, circulating co-metabolites, and bile acids gain prominence at different points of the AD continuum? How do these shifts modify pathology? Microbial production is only the first determinant. Host conversion, clearance, barrier integrity, and tissue-specific receptor distribution shape brain exposure and cellular response. Together, these processes provide a biological explanation for the relative ordering reported across clinical groups. Terminology is used according to biological origin. SCFAs and bacterial indole derivatives are treated as microbial metabolites, whereas TMAO is a host–microbial co-metabolite generated through microbial TMA formation followed by hepatic oxidation. Primary bile acids and most kynurenine-pathway metabolites are host-derived, secondary bile acids are microbially transformed host metabolites, and LPS and bacterial extracellular vesicles are discussed as non-metabolite microbial products.

### 3.1. Early Loss of Metabolic Resilience

#### 3.1.1. SCFA-Dependent Homeostasis

SCFAs connect microbial fermentation with epithelial, immune, and cellular metabolism. Acetate, propionate, and butyrate signal through G protein-coupled receptors, inhibit histone deacetylases, support epithelial junctions, and regulate immune activity [15,52]. These pathways also shape microglial maturation and inflammatory function [52,53]. Compound identity, dose, compartment, receptor distribution, and disease context determine the direction of the response [15,52]. A defined SCFA mixture, for example, increased microglial activation and motor deficits in α-synuclein-overexpressing mice [54]. Reduced fecal SCFAs therefore identify a change in the measured pool, not a uniform biological effect.

Microglial metabolism responds directly to acetate. Germ-free mice showed epigenetic changes in microglial metabolic genes, increased mitochondrial mass, and respiratory-chain dysfunction. Acetate restored metabolic fitness and modified microglial phagocytosis during neurodegeneration [53]. Acetyl-CoA synthetase 2 (ACSS2) links acetate availability to chromatin regulation. Acetate replenishment increased histone acetylation, glutamate-receptor expression, synaptic plasticity, and cognition in 5×FAD mice in an ACSS2-dependent manner [55]. SCFAs also strengthened the astrocyte-neuron glutamate–glutamine shuttle and the supply of glutathione precursors, thereby reducing neuronal oxidative injury in experimental systems [56].

Direct glial metabolism does not account for the entire SCFA response. In male APPPS1-21 mice, propionate reduced retinoic acid receptor-related orphan receptor gamma t (RORγt)-positive CD4 T cells and interleukin-17 (IL-17) secretion. IL-17 correlated with reactive astrocytosis, and cytokine depletion showed that reduced astrocyte reactivity and amyloid-β (Aβ) plaques depended on this pathway [57]. Gut bacterial depletion likewise lowered IL-17A-expressing T cells, brain inflammation, and Aβ; Il-17a deficiency abolished these effects [58]. Peripheral immune signaling therefore provides a second route from microbial fermentation to glial and amyloid pathology.

A commentary placed the propionate and IL-17 findings within a broader gut–brain–immune framework but added no independent experimental replication [59]. The evidence in this subsection therefore supports immune mediation of SCFA effects more strongly than a fixed reciprocal sequence.

#### 3.1.2. Receptor-Specific Indole Signaling

The microbiota regulates three major branches of tryptophan metabolism that generate indole derivatives, kynurenines, and serotonin [60]. The kynurenine branch includes 3-hydroxykynurenine, quinolinic acid, and kynurenic acid, which have different biological effects [60,61,62]. Their products differ in receptor affinity, redox activity, and tissue access [63]. Total tryptophan turnover therefore gives little information about the biological direction of the resulting signal.

Individual indoles recruit distinct cellular programs. Indole-3-lactic acid reduced soluble Aβ in 5×FAD mice through an aryl hydrocarbon receptor (AhR)-dependent response involving microglia and astrocytes [64]. Indole-3-propionic acid (IPA) crossed the blood–brain barrier and engaged neuronal pregnane X receptor (PXR); blocking PXR or disrupting its microbial synthesis abolished the cognitive and amyloid-related effects [65]. Indole-3-acetic acid (IAA) reduced microglial C-C chemokine receptor type 4 (CCR4) expression and synaptic engulfment in AD models. Circulating concentrations were lower in patients and correlated inversely with cognitive impairment [66].

AhR connects several of these responses. Indole, IAA, and IPA increased AhR activity and suppressed nuclear factor kappa B (NF-κB) signaling, NOD-like receptor family pyrin domain-containing 3 (NLRP3) inflammasome assembly, and inflammatory cytokine release in APP/PS1 mice [67]. Neuronal AhR activation also increased neprilysin transcription and Aβ-degrading activity [68]. A broader review links 5-hydroxyindole-acetic acid and kynurenic acid with metalloproteinases involved in cerebral Aβ clearance [69].

Multi-metabolite experiments require narrower interpretation. *Akkermansia muciniphila* administration increased IAA, tryptophan, acetate, and other metabolites in amyloid precursor protein/presenilin 1 (APP/PS1) mice. The shifts accompanied stronger AhR-related signaling, lower neuroinflammation, and less Aβ deposition [70]. IAA therefore forms part of a broader metabolite response whose individual contributions remain unresolved.

### 3.2. Expansion of Metabolic Injury

#### 3.2.1. Circulating Toxic Metabolites

Imidazole propionate (ImP) combines a human exposure signal with experimental vascular and neuronal effects. Among cognitively unimpaired adults, higher plasma ImP was associated with poorer cognition and biomarkers of AD and related dementias in cross-sectional and longitudinal analyses. Chronic exposure aggravated pathology in mice, impaired brain endothelial integrity, and promoted tau phosphorylation in primary neurons. Glycogen synthase kinase 3 beta (GSK3β) inhibition blocked the neuronal effect [71]. Independent structural work shows that GSK3β phosphorylation can catalyze tau assembly into filaments resembling those found in AD brains [72].

The host–microbial co-metabolite TMAO reaches tau pathology through more than one route. TMAO bound hypoxia-inducible factor 1 alpha (HIF1α), inhibited its signaling, increased oxidative stress, and promoted tau phosphorylation in cellular and P301S models [73]. A separate metabolite screen identified TMAO, indoxyl sulfate, and several other microbiome-dependent compounds as promoters of tau seeding. Systemic administration worsened cognition and tau pathology in mice [74].

Plasma TMAO was higher in participants with MCI. In rats, chronic choline exposure reduced inhibitory Ser9 phosphorylation of GSK3β and lowered proteins involved in synaptic plasticity. Blocking TMAO production or GSK3β prevented these deficits [75]. Human association and experimental pathway evidence therefore converge on TMAO exposure, although the GSK3β route has not been tested during human progression.

Kynurenine products can act in opposite directions. Oral *Porphyromonas gingivalis* exposure increased 3-hydroxykynurenine in serum and hippocampus. This metabolite suppressed B-cell lymphoma 2 (BCL2) and promoted neuronal apoptosis in mice and cell models [61]. In another AD mouse model, microbial modulation increased hippocampal kynurenic acid. This increase was associated with metabolic changes consistent with enhanced neuronal fatty acid oxidation, reduced lipid accumulation, and suppressed microglial activation [62]. Biological direction depends on the metabolite branch and experimental context, not on kynurenine metabolism as a single category.

#### 3.2.2. Bile Acid and Lipid Remodeling

Human evidence supports redistribution of the circulating bile acid pool. Primary cholic acid is often lower, whereas several conjugated and secondary species are higher in dementia. Longitudinal studies support this pattern, although individual bile acids vary across cohorts [76]. Serum profiling detected stage-related and sex-related differences. Selected changes appeared before clinical AD in men and improved predictive models [77]. A small cerebrospinal fluid study found hydroxybutyrate and bile acid differences among controls, MCI due to AD, and dementia [78]. These studies establish remodeling more firmly than a microbial or receptor-level cause in patients.

Takeda G protein-coupled receptor 5 (TGR5) experiments expose the importance of disease stage and cell identity. Early in AD mice, deoxycholic acid and neuronal TGR5 increased. TGR5 recruited a STAT3–APH1–γ-secretase pathway and promoted amyloidogenic APP processing in excitatory neurons [79]. During middle and late stages, TGR5 declined in medial septal cholinergic neurons. Local activation increased cholinergic activity, hippocampal neurogenesis, and cognition through the medial septal-to-dentate gyrus circuit [80]. Different cell populations and disease windows therefore redirect the same receptor pathway.

Lipid effects are equally molecule-specific. A *Bacteroides ovatus*-associated lysophosphatidylcholine (LPC) activated G protein-coupled receptor 119 (GPR119), lowered long-chain acyl-CoA synthetase 4 (ACSL4) expression, and restrained ferroptosis in a 5×FAD model. Fecal and serum LPC levels were also lower in individuals with AD [81]. Integrated serum and brain metabolomics linked gut microbial changes with glycerophospholipid disruption, glial activation, and neuroinflammation in APP/PS1 mice [82]. Conversely, host-derived 27-hydroxycholesterol disturbed microbial composition, lowered fecal SCFAs, and damaged intestinal junctions [83]. Lipid remodeling can thus transmit gut-to-brain effects or carry brain and host metabolic stress back to the intestine.

### 3.3. Barrier Failure and Neuroimmune Amplification

Barrier integrity determines tissue exposure to microbial products and metabolites. Mice without a gut microbiota had greater blood–cerebrospinal fluid barrier permeability and disorganized tight junctions. Recolonization or SCFAs restored junctional organization, altered microglial phenotype, and reduced Aβ in App knock-in mice [84]. Intestinal epithelial Dicer1 deletion changed bacterial abundance, increased low-density lipoprotein receptor-related protein 1 (LRP1) and ATP-binding cassette subfamily B member 1 (ABCB1) at the blood–brain barrier, and lowered cerebral Aβ [85]. This epithelial experiment links intestinal regulation with cerebral Aβ transport.

M-cell depletion changed the gut community and reduced colonic inflammation, amyloid accumulation, microglial dysfunction, and memory impairment in 5×FAD mice [86]. LPS is a bacterial structural component, not a metabolite. Its blood and brain levels are elevated in AD, while experimental exposure activates innate immunity and AD-related pathology [87]. LPS-bearing vesicles crossed the blood–brain barrier, activated microglial Piezo1, and triggered C1q-C3-dependent synaptic pruning [88]. Commensal vesicles also reversed the less inflammatory microglial state of germ-free AD mice and increased amyloid pathology [89].

Metabolites shape the response after exposure. *Bacteroides fragilis* produced 12-hydroxy-heptadecatrienoic acid and prostaglandin E2. Both lipids activated microglia and recruited neuronal CCAAT/enhancer-binding protein beta (C/EBPβ) and asparaginyl endopeptidase, followed by Aβ and tau pathology in mice [90]. Microbial gamma-aminobutyric acid (GABA) has been linked to mucin and tight-junction regulation, although its AD-specific mechanism remains inferential [91]. A mechanistic review integrates microbiota-sensitive microglial responses with neuroinflammation and synaptic dysfunction [92]. A knowledge-driven network prioritized links among microbial metabolites, microglial genes, and AD phenotypes, with SCFAs ranking highly. These computational relationships require experimental confirmation [93].

Human data capture this interface as a combined signature. Fecal microbial features correlate with circulating LPS, endothelial markers, cytokines, amyloid, phosphorylated tau, and neurodegeneration measures [94]. Cross-sectional sampling cannot order them. After barrier crossing, gastric *Helicobacter pylori* vesicles activated complement component 3 (C3)–C3a receptor (C3aR) signaling across astrocytes, microglia, and neurons as well as aggravated amyloid in mice [95]. This is complement amplification by a structural product, not a metabolic pathway.

### 3.4. Temporal Dynamics and Reciprocal Feedback

Different metabolite classes follow different production and handling routes. Diet, antibiotics, and microbial metabolism directly influence SCFA availability in the gut and other organs [96]. TMAO requires microbial and host conversion. Enterobacteriaceae reduce TMAO to trimethylamine, which enters the circulation and undergoes hepatic reoxidation [97]. Kidney function further changes systemic exposure. TMAO was not associated with incident dementia in a large population cohort overall, but the association emerged among participants with impaired renal function [98].

Bile acids pass through a longer host–microbial circuit. The liver synthesizes primary species, enterohepatic circulation recycles them, and microbial enzymes generate secondary forms. Diet, antibiotics, and intestinal transit reshape the pool [99]. These routes make unequal stage prominence biologically plausible. They do not prove that SCFAs, TMAO, and bile acids change in a fixed order within the same patient.

Established brain pathology can then alter the metabolic source. Ventricular Aβ injection changed the mouse gut microbiota after four weeks, damaged colonic structure, and suppressed cholinergic anti-inflammatory signaling [100]. In 5×FAD mice, amyloid pathology reorganized the colonic immune compartment and altered the distribution of gut-associated immune cells in the brain and meninges [101]. The intestinal ecosystem is therefore both a source of signals and a target of cerebral pathology.

Oxidative stress can reinforce this loop. Dysbiosis-related oxidative and inflammatory signals can weaken intestinal and brain barriers, expanding peripheral exposure and renewing innate immune activation [102]. Reviews consequently describe the relationship between gut dysbiosis and AD pathology as bidirectional [103]. Longitudinal network analysis supplies an ecological example. During progression in mice, the microbial network shifted from scale-free to random organization and reached marked disequilibrium at the late stage of progression. Functional modeling implicated a hub taxon in quinolinic acid synthesis [104].

**Figure 1:**
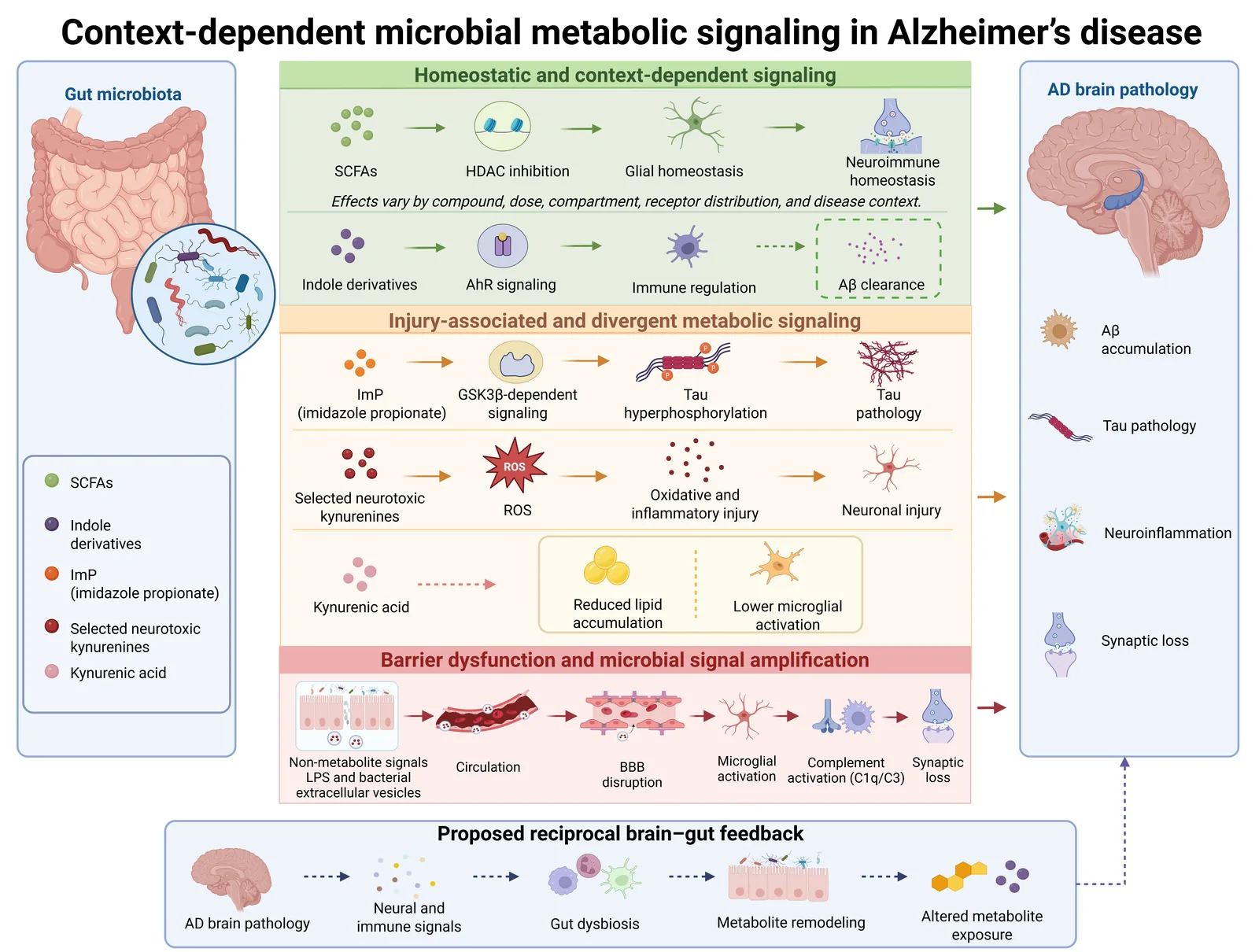
Context-dependent microbial metabolic signaling in Alzheimer’s disease. The figure integrates evidence from human studies and distinct experimental systems rather than depicting a single continuous causal pathway. Solid arrows denote relationships tested within the same experimental model through supplementation, depletion, receptor or pathway manipulation, or rescue. Dashed arrows denote associations or mechanisms synthesized across separate studies. Colors distinguish metabolite classes and pathway modules for visual clarity and do not represent quantitative effect size. SCFAs and indole derivatives illustrate context-dependent homeostatic and neuroimmune signaling, whereas ImP and selected kynurenine-pathway metabolites are linked to tau-related, oxidative, inflammatory, or neuronal effects. LPS and bacterial extracellular vesicles are non-metabolite microbial products. The barrier pathway summarizes representative experimental evidence and does not imply that all microbial products follow an identical sequence. The reciprocal brain–gut pathway is a proposed model assembled from multiple studies rather than a confirmed sequential process in human AD. Abbreviations: AD, Alzheimer’s disease; Aβ, amyloid-β; SCFAs, short-chain fatty acids; HDAC, histone deacetylase; AhR, aryl hydrocarbon receptor; ImP, imidazole propionate; GSK3β, glycogen synthase kinase 3 beta; ROS, reactive oxygen species; LPS, lipopolysaccharide; BBB, blood–brain barrier. Figure 1 was created with BioRender.com.

SCFA homeostasis, indole signaling, barrier amplification, and brain-to-gut feedback recur across experimental systems. Human studies establish stage-associated patterns, while animal and cell models resolve receptor and pathway mechanisms. The principal uncertainty lies in temporal translation because animal age does not map directly onto subjective cognitive decline, mild cognitive impairment, or dementia. Host genetic and physiological context can further shift the point at which a metabolic disturbance becomes clinically consequential. These context-dependent microbial metabolic pathways and their reciprocal interactions are summarized in Figure 1.

## 4. Apolipoprotein E4 and Metabolic Susceptibility

AD susceptibility is polygenic. Large genetic studies place apolipoprotein E (APOE) alongside TREM2, CLU, ABCA7, SORL1, BIN1, and other loci that converge on microglial function, lipid transport, and endosomal trafficking [105]. APOE4 has particularly close links to lipid handling and cellular metabolism [106,107]. These functions intersect with the absorption, distribution, and tissue response to gut-derived compounds, making APOE4 a focused model of genetically modified metabolic susceptibility.

Genetic background can influence microbial metabolic regulation at two levels. It shapes microbial ecology and host metabolism before exposure occurs. It also determines barrier access and cellular responses after circulating compounds reach their target tissues. Evidence is strongest for SCFA-associated ecology, whole-microbiota perturbation, and APOE4-dependent vascular and immune responses. This distinction separates variation in metabolite production from variation in biological effect. Comparable fecal or circulating concentrations can therefore carry different pathological implications across genetic backgrounds.

### 4.1. APOE4 Shapes the Microbial and Metabolic Context

Human data connect APOE4 to microbial metabolism most directly through dietary fibre and SCFAs. A cross-sectional study spanning subjective cognitive impairment, mild cognitive impairment, and normal cognition measured dietary intake, fecal microbial composition, and SCFAs in feces and serum. APOE4 carriers consumed less fibre, and genotype groups differed in microbial diversity and SCFA profiles. Associations linking fibre intake, cognition, and microbial metabolism were stronger in noncarriers. Fecal and serum measurements capture different stages of microbial production, intestinal absorption, and host clearance. Genotype-related patterns across both compartments therefore indicate altered host–microbial coupling. The cross-sectional design cannot establish whether the microbial profile preceded cognitive change [108].

Sex modified the APOE-associated microbial pattern in young EFAD mice. Fecal communities differed between APOE3- and APOE4-expressing animals, with the clearest genotype-associated separation in females [109]. Sex therefore alters the microbial environment in which APOE-dependent metabolic signals are generated.

Experimental manipulation strengthens the host-to-microbiota direction. In tauopathy mice expressing human APOE isoforms, germ-free rearing and antibiotic depletion reduced gliosis, tau pathology, and neurodegeneration. The magnitude varied according to APOE isoform and sex [110]. The experiment establishes a genotype-dependent effect of whole-microbiota perturbation. It does not attribute the response to a single bacterial metabolite.

APOE-dependent metabolic variation extends beyond the intestine. Prospective human data linked genetic predisposition, plasma metabolites, and dietary patterns to dementia risk [111]. APOE-stratified multi-omics separated genotype-specific signals from mitochondrial, inflammatory, and lipid abnormalities shared across genotypes [112]. Aging humanized mice showed genotype-dependent differences in brain amino acids, acylcarnitines, phospholipids, and sphingomyelins [113]. In human cohorts, sex and APOE status modified fatty-acid and acylcarnitine associations with cognitive decline [114]. Network analysis also identified a phosphatidylcholine-centred profile in APOE4 carriers [115]. Blood and brain metabolomes integrate dietary input, hepatic processing, organ clearance, and tissue turnover. Their genotype-dependent patterns define the systemic and cerebral components of metabolic susceptibility.

Targeted plasma profiling further showed that oxylipin and endocannabinoid pathways varied across disease stages and between sexes. APOE-stratified analyses linked bile acids, lipid-peroxidation products, and cortisol with metabolic profiles associated with resilience or conversion from mild cognitive impairment to AD [116]. This connects genotype and sex with metabolic variation during clinical transition.

### 4.2. Evidence for Reverse Metabolic Regulation

Direct evidence that a defined gut-derived metabolite regulates APOE4 expression or signaling in vivo remains limited. Current studies instead place microbial metabolism and APOE-related lipid processing within the same physiological system [117,118]. APOE-related proteins such as ATP-binding cassette transporter A1 (ABCA1), low-density lipoprotein receptor (LDLR), and low-density lipoprotein receptor-related protein 1 (LRP1) provide potential interfaces through which lipid-active metabolites could modify APOE-dependent responses. These findings are therefore considered as response-modifying interfaces rather than established metabolite–APOE4 causal pathways.

A pathway-level connection has been demonstrated for microbial bile acid metabolism. In SAMP8 mice, polysorbate 80-induced dysbiosis increased secondary bile acid-producing bacteria and raised deoxycholic acid in the serum and brain. Fecal microbiota transplantation reproduced cognitive and inflammatory abnormalities, while deoxycholic acid promoted microglial senescence-associated secretory responses through ABCA1-dependent lysosomal cholesterol transport [119]. The experiment establishes a microbial bile acid–ABCA1 route without testing APOE4 dependence.

Microbial inflammatory products reveal a related form of genotype-dependent responsiveness. Human iPSC-derived microglia carrying APOE3/3 or APOE4/4 responded to LPS plus interferon-γ with PI3K-AKT-mTOR activation and blocked autophagic flux. Aβ42 induced greater lysosomal membrane permeabilization in APOE4/4 cells and was accompanied by altered amino-acid metabolism [120]. LPS is a bacterial structural product rather than a metabolite. The experiment therefore demonstrates greater inflammatory and lysosomal vulnerability in an APOE4 background. It does not demonstrate metabolite-driven regulation of APOE4.

### 4.3. APOE4-Dependent Tissue Responses

Host lipid and energy handling provide the clearest response layer. APOE4 impaired cholesterol handling and myelination in human tissue, stem-cell models, and targeted-replacement mice [121]. Aged APOE4 mice also showed lower hippocampal acetyl-CoA, ATP, and citrate synthase activity [122]. Stable-isotope tracing revealed increased aerobic glycolysis, reduced pyruvate entry into the tricarboxylic acid cycle, and lower oxidative flexibility [123]. Human cerebrovascular lipidomics linked APOE4 to higher phosphatidylethanolamine and lower sphingomyelin [124]. Brain eicosanoid profiles and cellular multi-omics added genotype-dependent inflammatory lipid and amino-acid patterns [125,126]. These changes constrain how neural cells store, oxidize, and signal through metabolites that reach the brain.

Circulating gut-derived metabolites and microbial products next encounter the neurovascular interface. Human imaging detected greater cortical blood–brain barrier permeability in APOE4 carriers, including cognitively normal amyloid-negative individuals [127]. In biomarker-confirmed AD, insulin resistance showed its strongest interaction with barrier permeability in APOE4 homozygotes [128]. A meta-analysis of APOE4 target-replacement mice found a consistent reduction in cerebral blood flow, while vascular morphology showed a non-significant and method-dependent trend [129]. Peripheral vascular stress produced the same genotype gradient. Monomeric C-reactive protein caused greater CD31-associated cerebrovascular injury in APOE4 mice [130]. Circulating sPDGFRβ was elevated in cognitively impaired carriers [131]. Blood-pressure variability also tracked white-matter injury preferentially in carriers [132]. APOE4 thus changes both access to neural tissue and the vascular consequences of circulating exposure.

Once gut-derived signals reach neural tissue, immune cells add a second layer of genotype-dependent processing. A terminally inflammatory microglial state expanded with age and APOE4 burden in longitudinal mouse data and appeared in human AD cortex [133]. Adaptive immune composition also changed across mild cognitive impairment and AD in an APOE-dependent manner [134]. Relationships among immunoglobulin A, cognition, inflammation, and neuropathology were concentrated in noncarriers [135]. Prostaglandin and isoprostane associations with Tau biomarkers likewise differed across APOE strata, although they did not predict conversion from mild cognitive impairment to dementia [136]. APOE4 therefore redirects inflammatory processing across cell types, metabolic states, and stages of pathology. These vascular, lipid, and immune findings define an APOE4-dependent response context, but most were not designed to test mediation by a gut-derived metabolite.

### 4.4. Implications for Variable Metabolic Timing

APOE4 can shift metabolic trajectories at three points. It can alter the ecological and dietary context that shapes SCFA exposure. It can change systemic lipid and energy metabolism before a microbial product reaches the brain. It can also modify vascular entry and glial handling after exposure. Age, sex, diet, medication, and hepatic or renal function act on the same sequence. APOE4 shifts the transition threshold together with these host factors.

APOE4 may alter metabolic trajectories by modifying microbial ecology, systemic lipid handling, vascular access, and cellular responses. Direct evidence is strongest for genotype-associated microbial and metabolomic differences, whereas regulation of APOE4 by a defined gut-derived metabolite remains unproven. Longitudinal studies combining APOE genotype, microbial pathway capacity, compartment-specific metabolites, organ function, and AD biomarkers are required to test this model.

## 5. Precision Metabolic Intervention and Clinical Translation

The stage-associated profiles described earlier do not translate directly into treatment. Intervention requires a controllable source, a measurable exposure, and a biological response that can be tracked after treatment. This shifts the therapeutic objective from broadly restoring a “healthy” gut microbiota to correcting a defined microbial metabolic pathway in a selected patient population. Human studies have established that diet and probiotics can alter microbial and metabolic readouts. Experimental models provide stronger evidence for individual pathways. Few studies, however, have followed the entire sequence from microbial function to an AD biomarker and then to clinical outcome. The translational task is to connect these levels within one design.

### 5.1. Actionable Metabolic Targets

#### 5.1.1. Microbial Generation and Substrate Supply

Metabolite production begins with substrate availability and microbial pathway capacity. In adults at risk of AD, a controlled crossover study found that ketogenic and low-fat diets produced distinct changes in the microbiome, metabolome, and food-derived chemical profile [137]. Dietary exposure is therefore measurable upstream of the circulating metabolite. Fibre illustrates why the same input need not produce the same response. Its associations with gut microbial features, SCFAs, and cognition differed according to APOE4 status [108]. Chemical structure, fermentability, and the baseline microbial community further determine which organisms can use fibre and which products accumulate [138].

Taxonomic abundance alone cannot define this intervention level. Different organisms may encode the same reaction, while closely related strains can differ in metabolic output. Genome-scale community models offer a route from metagenomic content to predicted substrate use and metabolite exchange [139]. Such predictions still require experimental confirmation, but they can identify whether treatment failure reflects insufficient substrate, absence of the required enzyme, or competition within the community. A substrate-directed trial should therefore measure dietary intake, pathway genes, and the target metabolite together.

#### 5.1.2. Host Conversion, Clearance, and Barrier Exposure

Microbial release does not equal tissue exposure. Intestinal absorption, hepatic conversion, renal clearance, and barrier transport reshape the signal before it reaches the brain. Increased intestinal bile acid absorption raised systemic and cerebral bile acid exposure and contributed to cognitive impairment in an aging model [140]. This places the transporter and enterohepatic cycle beside microbial bile acid transformation as potential control points. The same reasoning applies to trimethylamine N-oxide. Its plasma concentration integrates dietary precursor intake, microbial trimethylamine production, hepatic oxidation, and kidney function. Lowering a microbial precursor and lowering circulating exposure are related, but they are not interchangeable therapeutic endpoints.

#### 5.1.3. Receptor and Downstream Signaling

Receptor engagement supplies a downstream readout. Multi-omics screening has mapped candidate interactions between gut microbial metabolites and the G protein-coupled receptor repertoire in AD, creating testable metabolite–receptor pairs [141]. Experimental studies provide more developed examples. Indole-3-lactic acid reduced amyloid pathology through aryl hydrocarbon receptor signaling [64]. Indole-3-propionic acid improved cognitive and pathological measures through pregnane X receptor-dependent responses [65]. These pathways justify targeted assays in intervention studies. They do not require the full mechanistic discussion to be repeated here. The practical question is whether treatment changes the intended metabolite in the relevant compartment and engages its proposed response node.

**Figure 2:**
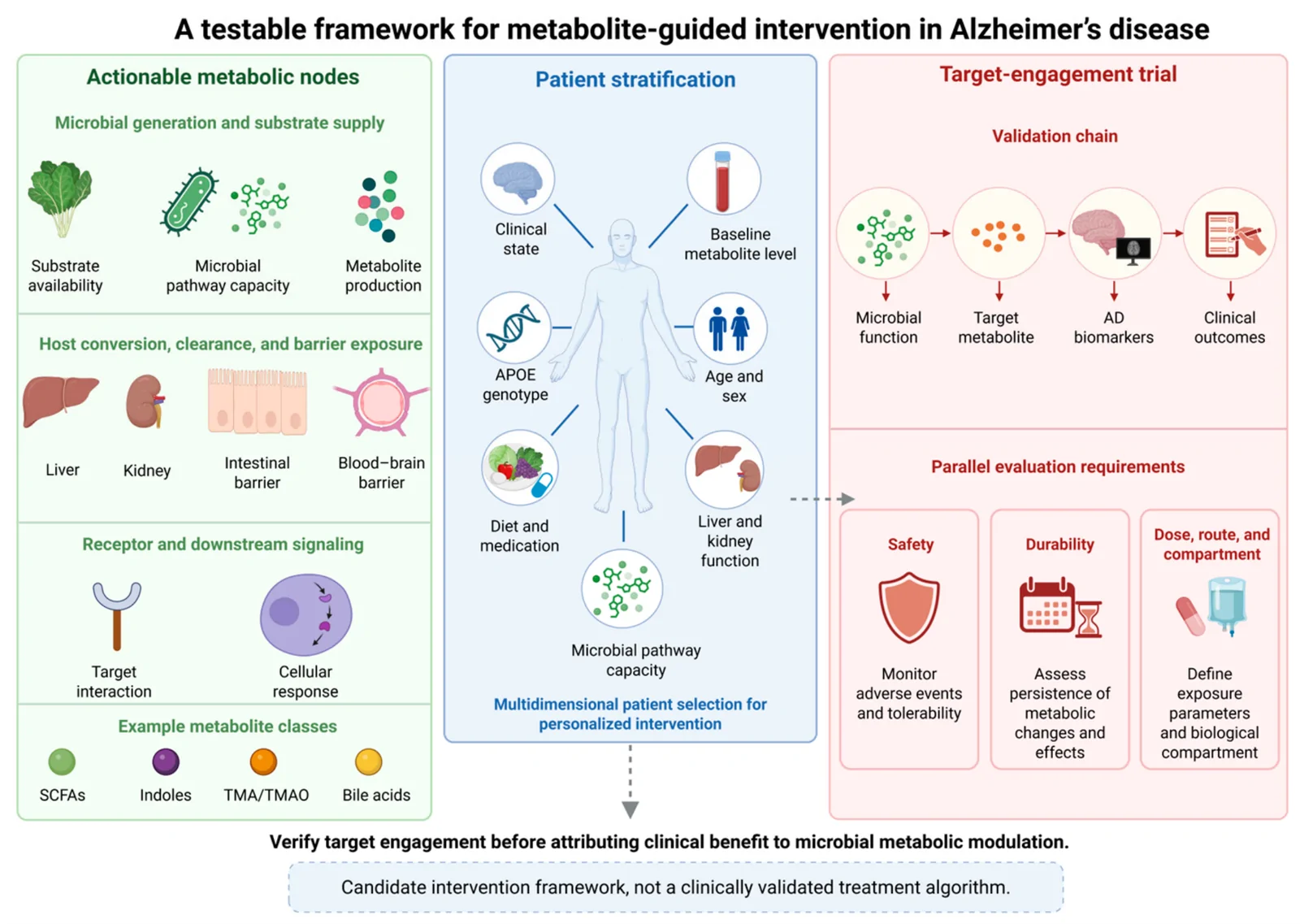
A testable framework for metabolite-guided intervention in Alzheimer’s disease. Actionable nodes include microbial substrate use and metabolite generation, host conversion and clearance, barrier exposure, and receptor-dependent cellular responses. Patient selection integrates clinical state, baseline metabolite level, APOE genotype, age, sex, diet, medication, liver and kidney function, and microbial pathway capacity. Target-engagement trials should verify sequential changes in microbial function, the target metabolite, AD biomarkers, and clinical outcomes while evaluating safety, durability, dose, route, and biological compartment. The framework is a candidate trial model and not a clinically validated treatment algorithm. Colors distinguish the major framework components for visual clarity; arrows indicate the proposed sequence from intervention targets to target engagement and clinical evaluation. Figure 2 was created with BioRender.com.

The three intervention levels are summarized in Figure 2. Microbial generation controls supply. Host processing determines exposure. Receptor and cellular responses define target engagement. The same figure extends these nodes into patient selection and a validation chain for clinical trials.

### 5.2. Evidence for Microbiome-Directed Interventions

#### 5.2.1. Diet, Prebiotics, and Probiotics

Human intervention evidence remains concentrated in multidomain or small exploratory studies. An intensive lifestyle randomized trial in participants with mild cognitive impairment or early AD dementia reported improvement in several cognitive and functional measures, together with changes in the gut microbiome and plasma biomarkers [142]. Because diet, exercise, stress management, and support were delivered together, the trial cannot assign the clinical response to microbial metabolism. The controlled dietary study described above resolved microbial and metabolic responsiveness more directly, but it was not designed to establish disease modification [137]. A modified Mediterranean-style dietary intervention linked gut microbial changes with brain-related outcomes and provides a closer model for integrating diet, microbiome, and neural measurements [143]. Collectively, these studies show that human metabolic engagement can be measured. Mediation still needs to be tested prospectively.

Probiotic evidence is developing along a similar gradient. Supplementation in probable AD altered gut microbial and metabolic features, including butyrate and tryptophan-related measures [144]. Experimental work can trace the pathway further. A synbiotic selected for indole-3-lactic acid production increased this metabolite and reduced neuroinflammation, amyloid accumulation, and cognitive impairment in female 5×FAD mice through aryl hydrocarbon receptor signaling [145]. *Bifidobacterium breve* HNXY26M4 also increased SCFAs and accompanied reduced neuroinflammation and cognitive deficits in APP/PS1 mice [146]. Multi-strain probiotics improved cognitive and pathological measures through AKT/GSK3β-related signaling in senescence-accelerated mice [147]. The first two examples measured a microbial product. The third primarily establishes an intervention response and a downstream pathway.

Dietary animal studies broaden the candidate space but also expose sources of heterogeneity. Ketogenic and medium-chain triglyceride regimens altered the gut ecosystem and AD-related phenotypes [148]. Methionine restriction modified microbial tryptophan metabolism and increased indole-3-propionic acid in a sex-dependent manner [149]. Intermittent fasting provided a stronger mediation design by linking microbial remodeling to indole-3-propionic acid and testing pathway dependence [150]. Polyphenol supplementation [151], a Mediterranean diet-inspired formulation [152], and xanthoceraside [153] each changed microbial or metabolic readouts together with AD-related outcomes in animals. These studies nominate pathways for testing. Their complex exposures make a single-metabolite explanation inappropriate unless rescue, blockade, or loss-of-function experiments isolate that component.

#### 5.2.2. Microbiota Transfer and Live Biotherapeutics

Microbiota transfer asks whether a disease-associated community carries a transmissible biological effect. AD-associated *Bacteroides fragilis* activated microglia and promoted pathology in a neuronal C/EBPβ transgenic model, while bacterial and lipid mediators narrowed the candidate route [90]. Such experiments establish community-level causality more directly than cross-sectional human profiles. They do not automatically identify the metabolite responsible for the complete transplant phenotype.

Defined live biotherapeutics may improve reproducibility. Encapsulation and nasal delivery enhanced the performance of a *Bifidobacterium* intervention in APP/PS1 mice, illustrating that viability, delivery, and tissue access influence efficacy [154]. Sodium oligomannate produced sex-dependent changes in the gut microbiota, metabolites, cerebral amyloidosis, and reactive microglia [155]. The differing responses reinforce the need to measure metabolic delivery in each subgroup. Postbiotics offer another route because a defined microbial product may be easier to manufacture and dose than a living community [156]. Clinical development will still require compositional control, pharmacokinetics, durability, and safety. At present, microbiota transfer is most valuable for causal discovery, whereas metabolite-standardized preparations are more compatible with conventional target-engagement trials.

### 5.3. Precision Translation

#### 5.3.1. Stage and Biomarker Stratification

The metabolic differences observed across cognitive states can guide enrollment, but cross-sectional stages are not treatment windows. A trial should first identify the pathway it intends to change. Baseline metabolite concentration, precursor-to-product ratio, and microbial pathway capacity can then define metabolic eligibility. Fecal SCFAs vary with sex and amyloid status, demonstrating that a single concentration needs clinical and biological context [12]. Human studies in mild cognitive impairment and AD also differ in sampling, diagnostic criteria, intervention duration, and measured outcomes [47]. These sources of variation should become prespecified design variables rather than post hoc explanations.

A useful biomarker panel spans the intervention chain. Stool metagenomics can assess pathway capacity. Fecal and plasma measurements capture local production and systemic exposure. Cerebrospinal fluid or imaging biomarkers position the metabolic response relative to amyloid, tau, neurodegeneration, and inflammation. Clinical measures establish functional relevance. A personalized dietary-supplement trial protocol already illustrates how baseline gut microbial features can inform formulation and prospective evaluation [157]. Regulatory translation will also require analytical validation, reproducible specimen handling, and qualification of the proposed microbiome-derived biomarker for its intended use [158].

#### 5.3.2. Host and Microbiome Stratification

Metabolic eligibility is only one dimension of patient selection. APOE genotype and sex shape metabolic networks in AD [115]. Large human datasets further connect genetic predisposition, the plasma metabolome, Mediterranean dietary patterns, and later cognitive outcomes [111]. These findings support genotype-aware hypotheses without making APOE the sole allocation variable. Age, sex, medication, diet, liver and kidney function, and baseline disease state all modify exposure or tissue response. Microbial pathway capacity determines whether a substrate-directed intervention can generate its intended product.

Patient selection should therefore combine the target pathway with host handling. A participant with low circulating metabolite levels but preserved microbial production may require a different strategy from one lacking the relevant pathway genes. Reduced renal clearance can elevate a circulating marker without increased microbial synthesis. Barrier dysfunction can change neural exposure at the same plasma concentration. Multidimensional selection is not an added layer after treatment choice. It determines which intervention is biologically coherent for that patient.

#### 5.3.3. Trial Design and Target Engagement

A clinically informative trial must connect pathway delivery with disease-relevant responses. The validation chain begins with microbial function, proceeds through the target metabolite and AD biomarkers, and ends with clinical outcomes. Safety, durability, dose, route, and biological compartment require parallel evaluation. Reviews of microbiome-directed treatment in neurodegenerative diseases show that existing studies vary widely in intervention, disease state, background medication, and endpoint selection [159,160]. AD-focused therapeutic reviews likewise identify neuroinflammation as a plausible target but emphasize the gap between experimental manipulation and clinical implementation [161].

Repeated sampling is essential. An early taxonomic response may disappear while metabolic output persists, or composition may remain stable despite altered pathway activity. Pharmacodynamic success requires a predefined change in the target metabolite at an appropriate dose and in the relevant compartment. AD biomarker and clinical responses must then be tested against that change. Mediation analysis can determine whether metabolic engagement accounts for part of the treatment effect. Negative results also become interpretable. They can distinguish failure to deliver the pathway from failure of the engaged pathway to alter disease biology.

The current evidence supports a candidate framework rather than a validated treatment algorithm. Human trials have shown that gut microbial and metabolic states are modifiable. Animal studies identify several pathways with stronger causal resolution. The next advance will come from bringing these strengths into the same clinical design. Metabolite-guided therapy is pathway correction. Correction must be measured.

## 6. Comparative Metabolic Signatures of Gut Microbiota-Derived Metabolites Across Neurodegenerative Diseases

### 6.1. Shared Metabolic Vulnerabilities Across Neurodegenerative Diseases

The comparison of Alzheimer’s disease (AD), Parkinson’s disease (PD), and amyotrophic lateral sclerosis (ALS) indicates that gut microbial metabolism is not a uniform disease signal, but a set of partially overlapping metabolic functions whose biological effects depend on the vulnerable cell population, pathological protein, host metabolic state, and disease stage. Across the three disorders, the most recurrent evidence concerns short-chain fatty acids (SCFAs), tryptophan-related metabolites, intestinal barrier integrity, and broader amino-acid metabolism [162,163]. These shared features provide a useful framework for comparison, but they should not be interpreted as evidence that the same metabolite has the same direction of effect in every neurodegenerative disease.

SCFAs, including acetate, propionate, and butyrate, connect dietary substrate availability with epithelial integrity, immune regulation, and host energy metabolism. In AD, SCFA-related changes have been linked to microglial and astrocytic responses and to amyloid-associated phenotypes [52,164]. PD studies likewise report changes in SCFA-producing capacity and fecal SCFA concentrations, although the direction and functional interpretation vary among cohorts and experimental systems [54,162]. In ALS, the available human evidence is more heterogeneous: a recent-onset case–control study did not detect a significant overall difference in fecal SCFA concentrations, although disease subtype altered taxon–SCFA relationships and several butyrate-associated taxa were reduced [165,166]. Thus, the common feature is better described as altered microbial metabolic capacity rather than a universal depletion of SCFAs.

Tryptophan metabolism provides a second shared interface. Gut microorganisms contribute indole derivatives, whereas host metabolism generates kynurenine-pathway products; these branches can influence aryl hydrocarbon receptor (AhR) signaling, redox balance, immune activation, and neuronal function. Altered aromatic amino-acid metabolism has been documented in PD and ALS [166,167], while AD studies provide mechanistic evidence that individual indoles can exert distinct effects rather than producing a uniform “protective” or “harmful” signal [64,65,66,67]. This distinction is important for cross-disease interpretation: total pathway abundance or a broad class-level change cannot substitute for measurement of the individual metabolite and its relevant exposure compartment.

The intestinal barrier represents a further point of convergence. Altered microbial metabolism may change epithelial energy supply, mucus integrity, immune tone, and systemic exposure to microbial products. However, barrier dysfunction can also arise secondarily from altered diet, medication, intestinal transit, reduced mobility, or systemic inflammation. Consequently, a disease-associated metabolic signature may be both a contributor to pathology and a consequence of disease progression. The comparative evidence therefore supports a model of reciprocal host–microbiome interaction rather than a simple unidirectional gut-to-brain pathway.

### 6.2. Parkinson’s Disease: Microbial Metabolites, α-Synuclein Pathology, and Neuroinflammation

#### 6.2.1. Clinical and Metabolic Features of the PD Gut–Microbiome Interface

PD provides a particularly informative comparison with AD because gastrointestinal dysfunction, including constipation and altered intestinal motility, can occur before or alongside motor manifestations. This temporal relationship makes PD a useful model for examining whether gut metabolic alterations accompany prodromal α-synucleinopathy. Meta-analytic and metagenomic studies have identified reproducible functional changes in the PD gut microbiome, including altered carbohydrate-active enzyme and vitamin biosynthesis pathways, while fecal SCFAs and polyamines were reduced in a large multi-country analysis [168]. Importantly, the taxonomic contributors to similar functional deficits differed across geographic datasets, illustrating why metabolic functions may be more portable than individual taxa [168].

Evidence from prodromal and early α-synucleinopathy further supports the relevance of microbial metabolic remodeling before established motor disease. In individuals with idiopathic REM sleep behavior disorder, a clinical prodrome of α-synucleinopathy, and in early PD, microbial pathways related to fatty-acid metabolism and several vitamin biosynthetic pathways differed from controls after adjustment for relevant covariates [169]. These findings suggest that microbial metabolic remodeling may be detectable during early disease, but they do not establish that the metabolic changes precede or cause α-synuclein pathology.

#### 6.2.2. SCFAs and α-Synuclein-Associated Neuroinflammation

SCFAs illustrate the context dependence of microbial metabolites in PD particularly well. In α-synuclein-overexpressing mice, microbiota-derived SCFAs enhanced microglial activation and motor deficits, demonstrating that increasing a metabolite class that is often considered beneficial in intestinal physiology does not necessarily produce a neuroprotective effect in an α-synucleinopathy model [54]. At the same time, human PD cohorts have reported lower fecal SCFA concentrations and reduced abundance of several SCFA-producing organisms [168]. These observations are not necessarily contradictory: fecal depletion, systemic exposure, receptor signaling, microbial community structure, and disease-specific immune states represent different levels of the same metabolic system. Therefore, therapeutic restoration of SCFAs in PD should be evaluated as a target-engagement question rather than assumed to be beneficial from fecal measurements alone.

This distinction is supported by early interventional evidence. A small controlled prebiotic dietary study increased fecal SCFAs and improved gastrointestinal symptoms, with a trend toward improvement in motor severity; the study also observed reductions in several metabolites considered potentially unfavorable in PD [170]. An exploratory randomized double-blind study administered propionate, butyrate, and/or a prebiotic alongside standard PD therapy for six months and reported changes in clinical, immune, and barrier-related outcomes [171]. The modest sample size, adjunctive design, retrospective trial registration, heterogeneous response, and absence of disease-modification endpoints preclude efficacy conclusions. These findings should therefore be treated as hypothesis-generating evidence of metabolic target engagement.

#### 6.2.3. Aromatic Amino-Acid Metabolites and Proteolytic Microbial Metabolism

Beyond SCFAs, PD is associated with altered microbial proteolytic metabolism. In a large clinical cohort, plasma phenylacetylglutamine (PAGln), p-cresol sulfate/glucuronide, and indoxyl sulfate were higher in PD than in controls; after adjustment for age, sex, and medications, PAGln and p-cresol glucuronide were associated with motor symptom severity, and several aromatic amino-acid metabolites were associated with constipation [167]. Importantly, these metabolites were also correlated with specific gut microbial taxa, providing evidence that systemic metabolite phenotypes can retain a measurable relationship with microbial ecology. The study is observational, however, and therefore cannot determine whether the metabolites contribute to disease progression or reflect altered diet, transit, medication exposure, or disease-related physiology.

The clinical relevance of this axis is strengthened by experimental dietary modulation. In a pilot prebiotic intervention, fecal SCFAs increased while several potentially neuroactive or proteolysis-associated metabolites, including p-cresol sulfate and quinolinic acid, decreased [170]. Together, these findings suggest that PD may involve a shift in the balance between carbohydrate fermentation and proteolytic metabolism. This hypothesis should be tested longitudinally using paired dietary records, stool metagenomics, fecal and plasma metabolomics, and clinical measures of both motor and gastrointestinal function.

#### 6.2.4. Imidazole Propionate as a Disease-Specific Microbial Metabolite Axis

Recent mechanistic evidence provides a more disease-specific example of how a microbial metabolite may connect gut ecology with PD pathology. A 2025 study identified enrichment of *Streptococcus mutans* and its urocanate reductase (UrdA) in the gut microbiome of patients with PD, together with increased circulating ImP. In experimental systems, colonization with UrdA-producing bacteria increased systemic and brain ImP, produced dopaminergic neuronal loss, astrogliosis, microgliosis, and motor impairment, and aggravated α-synuclein pathology; administration of ImP reproduced key PD-like phenotypes, with mechanistic target of rapamycin complex 1 (mTORC1) activation implicated in the mechanism [172]. These findings are substantially stronger than a simple taxonomic association because they connect a defined microbial enzyme to a defined metabolite and then to pathological phenotypes. Nevertheless, the causal evidence is predominantly experimental, and the extent to which the UrdA–ImP pathway explains human PD heterogeneity remains to be established.

#### 6.2.5. Prodromal Disease, α-Synuclein Propagation, and Causality

The PD gut–brain relationship should therefore be considered within a temporal framework. Gastrointestinal dysfunction and microbial metabolic alterations may occur during prodromal α-synucleinopathy, while α-synuclein pathology itself can alter the enteric environment [169]. Experimental studies further show that fecal microbiota from PD donors can modify motor and neuroinflammatory phenotypes in α-synuclein models [54]. These observations are compatible with a bidirectional loop involving intestinal microbial metabolism, enteric neural signaling, α-synuclein aggregation, systemic immunity, and central microglial activation. They do not, however, establish a single initiating event. Future longitudinal studies should determine whether specific microbial functions or metabolites change before α-synuclein biomarker conversion and whether intervention at that stage changes subsequent motor or non-motor progression.

### 6.3. Amyotrophic Lateral Sclerosis: Gut–Immune–Muscle Metabolic Interactions

#### 6.3.1. Human Microbiome and Metabolic Heterogeneity in ALS

ALS differs fundamentally from AD and PD in that motor-neuron degeneration is accompanied by prominent systemic metabolic abnormalities, skeletal-muscle wasting, nutritional challenges, and marked clinical heterogeneity. Consequently, the relevance of gut microbial metabolism may extend beyond a conventional gut–brain pathway to interactions among the gut, immune system, motor neurons, and skeletal muscle. Human studies have reported altered microbial composition and reduced abundance of some butyrate-producing taxa, but findings are not uniform across cohorts [165,166]. A 2025 systematic review of human studies similarly concluded that alterations in gut microbial composition and several metabolic features have been reported, while emphasizing small sample sizes, methodological heterogeneity, and limited longitudinal evidence [173].

This heterogeneity is biologically important rather than merely methodological. ALS comprises different clinical phenotypes and genetic subtypes, and experimental studies indicate that microbiome effects can be subtype-dependent. For example, depletion of the microbiome worsened disease in SOD1 models but had a different effect in C9orf72 models, emphasizing that the same microbial perturbation cannot be assumed to have a uniform effect across ALS [174]. Human studies should therefore stratify by disease subtype, site of onset, nutritional status, medication exposure, and disease stage before interpreting a metabolite as a disease-specific biomarker.

#### 6.3.2. SCFAs, Butyrate-Producing Microbes, and the Gut–Muscle Axis

SCFAs are currently the most directly studied microbial metabolic axis in ALS. In patients studied shortly after symptom onset, overall fecal SCFA concentrations did not differ significantly from controls, although microbial composition and the relationships between individual taxa and SCFAs differed by disease status and ALS subtype; spinal-onset disease showed a trend toward lower propionate [165]. Other human data indicate reduced abundance of several dominant butyrate-producing organisms [166]. Taken together, these findings argue against describing ALS simply as a state of universal SCFA depletion. Instead, ALS may involve altered microbial capacity, altered substrate use, or altered host handling of microbial products, with the resulting metabolite exposure varying across patients.

The gut–muscle axis provides a plausible framework for interpreting these findings. Microbial metabolites can influence epithelial integrity, inflammatory signaling, substrate availability, and systemic energy metabolism, while skeletal muscle and mitochondrial metabolism are major determinants of functional reserve in ALS. However, evidence that gut-derived SCFAs directly alter motor-neuron survival in ALS is currently insufficient. The more defensible hypothesis is that microbial metabolites may modify systemic metabolic and inflammatory conditions that influence muscle maintenance and neuronal vulnerability. This distinction should be preserved in future intervention studies.

#### 6.3.3. Amino-Acid Metabolism, Energy Homeostasis, and Microbial–Host Crosstalk

Amino-acid metabolism is another potentially important bridge in ALS. The disease is associated with altered systemic energy balance and skeletal-muscle catabolism, while the gut microbiota can transform dietary amino acids into a wide range of bioactive products. Current ALS microbiome studies have reported alterations in microbial and metabolic features, but direct attribution of specific amino-acid metabolites to microbial production remains incomplete [173]. A small clinical fecal microbiota transplantation (FMT) report in two patients with advanced ALS described improvements in respiratory and motor-related measures accompanied by increases in several *Bacteroides* and *Faecalibacterium* species and changes in arginine- and branched-chain amino acid-related metabolites [175]. Because the study involved only two patients and lacked a controlled design, these findings should be considered hypothesis-generating rather than evidence of efficacy.

The same caution applies to the kynurenine and tryptophan pathways. These pathways are biologically relevant to neuroinflammation, redox balance, and excitotoxicity, but evidence specifically demonstrating that gut microbial production of individual kynurenine metabolites drives motor-neuron degeneration in ALS remains limited. Future studies should distinguish microbial indole production from host kynurenine metabolism and measure both pathways in parallel. This would help determine whether altered tryptophan metabolism represents a microbial signal, a host response, or a secondary consequence of systemic disease.

#### 6.3.4. Barrier Dysfunction, Enteric Function, and Reverse Causality

ALS also illustrates why reverse causality must be explicitly considered. Dysphagia, altered food intake, constipation, reduced mobility, respiratory dysfunction, nutritional support, and medication use can modify intestinal transit, substrate availability, and microbial ecology [173,176]. Consequently, a cross-sectional difference in fecal metabolites may reflect the metabolic consequences of advanced disease rather than an upstream pathogenic signal. Longitudinal sampling beginning near diagnosis, together with detailed dietary and nutritional measurements, is therefore essential.

### 6.4. Cross-Disease Comparison and Precision Metabolite-Based Intervention

The expanded comparison reveals three different disease-specific configurations of the gut microbial metabolic interface. In AD, microbial metabolites can be linked to amyloid and tau biology through immune, vascular, and neuronal pathways. In PD, the strongest disease-specific signals currently center on interactions among intestinal dysfunction, α-synuclein pathology, neuroinflammation, and selected metabolites such as aromatic amino-acid products and ImP. In ALS, the emerging framework is broader and more systemic, involving immune regulation, energy homeostasis, skeletal muscle, and the enteric system. These differences argue against a universal “microbiome restoration” strategy.

The same metabolite may also have different biological consequences across diseases. SCFAs can support epithelial and immune homeostasis, yet experimental α-synucleinopathy models show that SCFAs can enhance microglial activation and motor pathology [54]. Conversely, human PD intervention studies suggest that increasing SCFA exposure may improve gastrointestinal outcomes and may have clinical relevance [170,171]. This apparent discrepancy emphasizes the importance of dose, metabolite identity, receptor distribution, tissue compartment, disease stage, and host inflammatory state. Therapeutic studies should therefore define the intended metabolic exposure rather than use a generic increase in “beneficial bacteria” as the endpoint.

For PD, candidate strategies include targeted modulation of proteolytic metabolism, the UrdA–ImP pathway, SCFA exposure, and other metabolites linked to intestinal inflammation and α-synuclein biology. For ALS, candidate approaches should prioritize patient stratification and metabolic support, with particular attention to SCFA-producing functions, amino-acid metabolism, barrier integrity, nutritional status, and the gut–muscle axis. In both diseases, mechanistic claims should require evidence that an intervention changes the microbial function, changes the predicted metabolite exposure, alters a disease-relevant biomarker or physiological pathway, and ultimately improves a clinical outcome.

Overall, PD and ALS should not be treated as brief extensions of the AD literature. They provide biologically distinct tests of whether microbial metabolism is a general feature of neurodegeneration or a disease-context-dependent mediator. The current evidence supports shared metabolic vulnerabilities but also reveals disease-specific pathways and different levels of causal confidence. Future studies should therefore integrate longitudinal metagenomics, targeted metabolomics, host genetics, nutritional and medication data, and disease-specific biomarkers. Such an approach will allow microbial metabolism to be evaluated not simply as a diagnostic signature, but as a measurable and disease-contextualized therapeutic target. Key gut microbial metabolic findings across AD, PD, and ALS are summarized in Table 1, whereas study-level evidence and methodological details are provided in Table 2.

**Table 1:** Summary of gut microbial metabolic findings across AD, PD, and ALS.

Metabolic Axis	Disease and Stage/Model	Compartment	Reported Pattern	Evidence Source	Key References
SCFAs/butyrate	AD; SCD/MCI-associated cohorts	Feces and plasma	Stage-associated differences in SCFA pools and producer abundance	Human observational	[18,21,44]
SCFAs/butyrate	AD; mouse models	Gut, plasma, and brain	Propionate or butyrate altered glial, inflammatory, and amyloid phenotypes; effects depended on compound and model	Animal/mechanistic	[57,164]
Indole derivatives	AD; mouse and cell models	Gut and brain	ILA, IPA, and IAA engaged distinct immune and amyloid-related pathways	Animal/cell	[64,65,66,67]
TMAO	AD; MCI and dementia	Serum or CSF	Higher TMAO was associated with MCI or AD-related clinical and biomarker measures	Human observational	[39,177]
Bile acids	AD; MCI and dementia	Serum and brain	Lower primary cholic acid and higher secondary DCA-family measures were associated with cognition or AD biomarkers	Human observational/longitudinal	[36,38]
Kynurenine metabolites	AD; mouse and cell models	Serum and hippocampus	3-hydroxykynurenine and kynurenic acid showed divergent biological effects	Animal/cell	[61,62]
SCFAs	PD; α-synuclein-overexpressing mice	Gut and brain	SCFAs enhanced microglial activation and motor pathology in this model	Animal/mechanistic	[54]
Aromatic amino-acid metabolites	PD; clinically diagnosed	Plasma and feces	Higher PAGln, p-cresol conjugates, and indoxyl sulfate were associated with severity or constipation	Human observational	[167]
SCFA-related ecology	ALS; recent onset	Feces	Overall SCFA concentrations did not differ significantly; taxon–SCFA relationships varied by disease and subtype	Human case–control	[165]
Butyrate-producing bacteria	ALS; clinically diagnosed	Feces	Several dominant butyrate-producing taxa were less abundant than in healthy controls	Human case–control	[166]
PD prodromal/early	RBD and early PD; stool	Feces	Altered microbial fatty-acid and vitamin/cofactor biosynthesis pathways; some changes remained after covariate adjustment	Human observational	[13,169]
Imidazole propionate	PD; patient samples and mouse models	Plasma, brain, gut	Higher ImP and UrdA; gut colonization or ImP administration produced PD-like pathology in mice and aggravated α-synuclein pathology	Human + animal/mechanistic	[172]
SCFAs/prebiotic intervention	PD; controlled pilot	Feces, urine, clinical	Prebiotic increased fecal SCFAs and improved gastrointestinal symptoms; motor improvement was exploratory	Human interventional	[170]
SCFAs/prebiotic	PD; randomized double-blind study	Blood, feces, clinical	Six-month changes in clinical, immune, and barrier-related outcomes were reported; efficacy and disease modification remain unconfirmed	Exploratory randomized human study	[171]
Microbial amino-acid metabolites	ALS; human studies	Feces/plasma	Altered amino acid-related pathways and metabolites reported; causal microbial contribution remains uncertain	Human observational	[173]
FMT-associated metabolites	ALS; 2-patient case report	Feces/plasma	Changes in *Bacteroides/Faecalibacterium* and arginine/BCAA-related metabolites accompanied clinical improvement	Human case report	[175]
Microbiome–immune signaling	ALS; experimental models	Gut/systemic/CNS	Microbiome effects differed by ALS model/genetic background, supporting subtype-specific responses	Animal/mechanistic	[174]

Abbreviations: AD, Alzheimer’s disease; ALS, amyotrophic lateral sclerosis; BCAA, branched-chain amino acid; CNS, central nervous system; CSF, cerebrospinal fluid; FMT, fecal microbiota transplantation; IAA, indole-3-acetic acid; ILA, indole-3-lactic acid; ImP, imidazole propionate; IPA, indole-3-propionic acid; MCI, mild cognitive impairment; PAGln, phenylacetylglutamine; PD, Parkinson’s disease; RBD, rapid eye movement sleep behavior disorder; SCFAs, short-chain fatty acids; SCD, subjective cognitive decline; TMAO, trimethylamine N-oxide; UrdA, urocanate reductase.

**Table 2:** Study-level evidence for gut microbial metabolites and related microbial products across AD, PD, and ALS.

Metabolite/Microbial Product	Biological Source	Compartment	Disease/Stage/Model	Cohort or Model Size	Analytical Platform	Direction/Principal Finding	Confounders/Adjustments	Evidence Level	Ref.
SCFAs (acetate, propionate, butyrate, isovalerate)	Microbial fermentation products	Feces; linked to CSF/plasma biomarkers	AD continuum; cognitively unimpaired/amyloid-defined and dementia participants	*n* = 287; 163 female + 86 male CU, 21 female + 17 male dementia; 65 A+	Fecal SCFA quantification + gut bacterial abundance profiling; regression/mediation analyses	Propionate, isovalerate and propionate-producing bacteria inversely associated with amyloid status; SCFAs associated with slower cognitive decline; sex-specific effects	Sex and amyloid status explicitly modeled; additional clinical/demographic covariates assessed	Human longitudinal cohort/observational	[12]
*Faecalibacterium prausnitzii*/butyrate-producing taxa	Microbial; butyrate-producing bacteria	Feces	AD-type dementia	NR	Gut microbiome profiling	Lower abundance of F. prausnitzii and other SCFA-producing taxa associated with poorer cognition/AD pathology	NR	Human observational	[21,22,23]
Bile acids	Host-derived, microbially modified co-metabolites	Serum; CSF/brain-related biomarkers	MCI and AD; longitudinal AD cohorts	NR	Targeted/untargeted serum bile-acid profiling	Lower primary cholic acid with higher secondary/bacterially modified bile-acid measures; associations with cognition and AD biomarkers	NR	Human observational/longitudinal	[36,37,38]
TMAO	Host–microbial co-metabolite	CSF; plasma/serum	MCI and AD	NR	Metabolomic/targeted TMAO measurement	Higher TMAO associated with MCI/AD-related clinical and biomarker measures; direction may depend on renal function	Renal function is a relevant modifier; exact model covariates NR	Human observational	[39,98]
Propionate	Microbial fermentation product	Gut/plasma; brain inflammatory pathway	APPPS1-21 AD mouse model	Male APPPS1-21 mice; NR	Experimental intervention; cytokine/immune and pathology assays	Propionate reduced RORγt+ CD4 T cells and IL-17; associated with reduced astrocyte reactivity and Aβ plaques; pathway dependence on IL-17	Sex/model background specified; experimental controls	Animal mechanistic	[57]
Butyrate	Microbial fermentation product	Gut, plasma, brain	AD mouse model/gut–brain amyloid pathology	Mouse group sizes reported separately across experiments	Experimental metabolite intervention + amyloid/inflammatory assays	Butyrate reduced Aβ secretion/accumulation and neuroinflammatory pathology in the cited model	Animal model, dose and treatment duration are model-specific	Animal mechanistic	[164]
Indole-3-lactic acid (ILA)	Microbial tryptophan metabolite	Gut/brain	AD mouse model	5×FAD mice; NR	Metabolite intervention + AhR/pathology assays	Reduced amyloidopathy through AhR-dependent signaling involving microglia and astrocytes	Experimental model/treatment conditions	Animal mechanistic	[64]
Indole-3-propionic acid (IPA)	Microbial tryptophan metabolite	Gut/brain	AD experimental models	Mouse model; NR	Metabolite intervention + PXR/amyloid/cognition assays	Crossed BBB and engaged neuronal PXR; disruption of IPA synthesis or PXR signaling abolished reported effects	Experimental model/intervention conditions	Animal mechanistic	[65]
Indole-3-acetic acid (IAA)	Microbial tryptophan metabolite	Circulation/brain; human association plus AD models	AD patients and experimental AD models	Human cohort size NR; animal model *n* NR	Metabolite measurement + CCR4/synaptic-loss assays	IAA reduced microglia-mediated synaptic loss in models; circulating IAA reported lower in patients and inversely related to cognitive impairment	NR for human adjustment set	Human observational + animal mechanistic	[66]
Microbial indoles (indole, IAA, IPA)	Microbial tryptophan metabolites	Gut/brain	APP/PS1 mice	APP/PS1 model; NR	Metabolite exposure + AhR/NF-κB/NLRP3 assays	Increased AhR activity and suppressed inflammatory signaling in the cited experimental model	Experimental model/treatment conditions	Animal mechanistic	[67]
Imidazole propionate (ImP)	Microbial histidine-derived metabolite	Plasma; brain/endothelial and neuronal experimental systems	Cognitively unimpaired adults with ADRD biomarkers + mouse/cell models	Human cohort size NR; animal/cell experiments	Targeted ImP measurement + mouse exposure + endothelial/neuronal assays	Higher plasma ImP associated with poorer cognition and ADRD biomarkers; chronic exposure worsened pathology and promoted tau phosphorylation in models	Longitudinal/cross-sectional human analyses; exact covariate set NR	Human observational + animal/cell mechanistic	[71]
3-Hydroxykynurenine	Tryptophan–kynurenine pathway metabolite; microbiota-influenced	Serum and hippocampus	P. gingivalis-exposed AD mouse/cell models	Animal/cell; NR	Metabolite measurement + animal/cell apoptosis assays	Increased 3-HK associated with BCL2 suppression and neuronal apoptosis	Experimental exposure conditions	Animal/cell mechanistic	[61]
Kynurenic acid	Tryptophan–kynurenine pathway metabolite	Hippocampus	AD mouse model	Animal model; NR	Metabolite/metabolic profiling + inflammatory and neuronal assays	Increased kynurenic acid associated with metabolic changes, reduced lipid accumulation and suppressed microglial activation in the cited model	Experimental model	Animal mechanistic	[62]
SCFAs	Microbial fermentation products	Gut/brain	Thy1-α-synuclein overexpressing PD mice; germ-free and colonized conditions	Mouse group sizes reported separately across experiments	16S rRNA profiling; fecal SCFA HPLC; germ-free/colonization and metabolite intervention	Defined SCFA mixture promoted microglial activation and motor deficits in α-synuclein-overexpressing mice; effects were genotype-dependent	Genotype, microbiota status and treatment condition controlled experimentally	Animal mechanistic	[54]
SCFAs/microbiome–SCFA relationships	Microbial fermentation products	Feces	Recent-onset ALS (<6–15 months after symptom onset)	*n* = 28: 16 ALS, 12 controls	16S rRNA gene V3–V4 sequencing; fecal SCFA HPLC	No significant overall SCFA concentration difference; subtype- and taxon-specific relationships, with a trend toward lower propionate in spinal ALS	Age, disease duration and clinical subtype considered; exact multivariable adjustment not established	Human case–control pilot	[165]
Aromatic amino-acid metabolites: PAGln, p-cresol sulfate/glucuronide, indoxyl sulfate	Microbial/host–microbial co-metabolites	Plasma; feces	Clinically diagnosed PD; mean Hoehn–Yahr stage ~3.2	*n* = 500: 250 PD, 250 controls; fecal microbiome data 154 new PD + 96 previously reported PD	Plasma LC-MS; fecal shotgun metagenomic sequencing	Higher PAGln, p-cresol sulfate/glucuronide and indoxyl sulfate in PD; PAGln and Pcg positively associated with motor severity; metabolites associated with constipation	Multivariable models adjusted for age, sex and medications; diet, BMI, comorbidities and creatinine assessed	Human case–control/observational	[167]
Butyrate-producing bacteria	Microbial taxa with butyrate-producing capacity	Feces	Clinically diagnosed ALS	139 stool samples: 66 ALS, 61 healthy controls, 12 neurodegenerative controls	Shotgun metagenomic sequencing	*Eubacterium rectale*, *Roseburia intestinalis* and total abundance of dominant butyrate-producing species were lower in ALS vs. healthy controls	Adjusted for age, sex and constipation; results robust to adjustment	Human case–control	[166]

Note: NR, not reported in the cited source or not sufficiently specified for reliable extraction. Association is not interpreted as causation. Sample sizes and analytical platforms were retained only when supported by the cited study.

## 7. Limitations and Research Priorities

This review has several limitations. It is a narrative synthesis rather than a systematic review or meta-analysis, and the literature search was centered on PubMed. Although reference lists and high-quality reviews were screened to identify additional studies, incomplete database coverage and publication bias remain possible. The evidence base is heterogeneous in diagnostic criteria, sampling procedures, analytical platforms, dietary assessment, medication exposure, and adjustment for age, sex, APOE genotype, intestinal transit, and liver or kidney function.

Most human microbiome and metabolite studies are cross-sectional and compare independent clinical groups. SCD and clinically defined MCI are not equivalent to biomarker-confirmed preclinical or prodromal AD, and group-level differences cannot reconstruct a within-person metabolic trajectory. Fecal, circulating, CSF, and brain measurements reflect different stages of production, absorption, conversion, clearance, barrier passage, and tissue use and are therefore not interchangeable. Animal age and transgenic pathology likewise do not map directly onto human clinical stages.

Causal confidence is strongest in experimental systems combining microbial depletion or transfer, metabolite supplementation, receptor or cytokine blockade, genetic manipulation, and rescue. Such designs remain uncommon in humans. Changes in community composition, cognition, or inflammation alone do not establish metabolite mediation. The temporal and intervention frameworks proposed here should therefore be treated as testable models rather than established patient-level trajectories or clinically validated treatment algorithms.

## 8. Conclusions

Gut microbial metabolism is a dynamic interface between intestinal ecology, host physiology, and neurodegenerative pathology. Across the AD continuum, fermentation-related signals are detectable in prodromal cohorts, while broader amino-acid, host–microbial co-metabolite, bile acid, and lipid abnormalities accompany cognitive impairment and dementia. Comparison with PD and ALS shows that these pathways are not disease-neutral: PD is characterized by a particularly prominent interaction among gastrointestinal dysfunction, α-synuclein pathology, neuroinflammation, and selected microbial metabolites, whereas ALS points toward a gut–immune–muscle and systemic metabolic interface with greater heterogeneity and weaker causal evidence.

Mechanistic evidence is strongest where defined metabolites are linked to receptors, cellular responses, or rescue experiments. In PD, recent evidence for microbial aromatic amino-acid metabolites, SCFA-related signaling, and the UrdA–imidazole propionate pathway provides increasingly specific metabolic hypotheses. In ALS, human studies support altered microbial ecology and metabolic relationships, but subtype effects, nutritional status, and reverse causality remain major limitations. These differences reinforce the central conclusion that shared microbial pathways do not imply shared therapeutic effects. Useful interventions will need to be stage-specific, compartment-aware, and matched to disease-specific biology.

Clinical translation now depends on completing the causal chain. Longitudinal studies should align repeated microbial functions and metabolite measurements with AD biomarkers and clinical outcomes. Intervention trials must show that the intended pathway changed, that the resulting exposure reached its target, and that biomarker and functional responses followed. Community composition or cognition alone cannot establish metabolic mediation. Comparisons with PD and ALS reinforce the same principle. Shared pathways do not guarantee shared effects because vulnerable cells, proteinopathies, anatomical origins, and drug exposures differ among diseases. Gut microbial metabolism offers a common language for neurodegeneration, but useful targets will be stage-specific, compartment-aware, and matched to the biology of the individual patient.

## Data Availability

No new data were created or analyzed in this study. Data sharing is not applicable to this article.
